# The effect of sequential combination of mirror therapy and robot-assisted therapy on motor function, daily function, and self-efficacy after stroke

**DOI:** 10.1038/s41598-023-43981-3

**Published:** 2023-10-06

**Authors:** Yen-Wei Chen, Kuan-Yi Li, Chu-Hsu Lin, Pei-Hsuan Hung, Hui-Tzu Lai, Ching-Yi Wu

**Affiliations:** 1 Department of Physical Therapy, College of Medical and Health Science, Asia University, NO.500, Lioufeng Rd., Wufeng, Taichung, 41354 Taiwan; 2 Department of Occupational Therapy and Graduate Institute of Behavioral Science, College of Medicine, Chang Gung University, No.259, Wenhua 1St Rd., Guishan Dist., Taoyuan City, 33302 Taiwan; 3 Department of Physical Medicine and Rehabilitation, Chang Gung Memorial Hospital at Chiayi, No.8, Sec. W., Jiapu Rd., Puzi City, Chiayi County 61363 Taiwan; 4 Department of Physical Medicine and Rehabilitation, Jiannren Hospital, No. 136, Nanyang Rd., Nanzi Dist., Kaohsiung City, 811504 Taiwan; 5 Department of Physical Medicine and Rehabilitation, LO-Sheng Hospital Ministry of Health and Welfare, No.794, Zhongzheng Rd., Xinzhuang Dist., New Taipei City, 24257 Taiwan; 6 Healthy Aging Research Center, Chang Gung University, Taoyuan, Taiwan; 7 Department of Physical Medicine and Rehabilitation, Chang Gung Memorial Hospital at Linkou, Taoyuan, Taiwan

**Keywords:** Rehabilitation, Stroke

## Abstract

Robot-assisted therapy and mirror therapy are both effective in promoting upper limb function after stroke and combining these two interventions might yield greater therapeutic effects. We aimed to examine whether using mirror therapy as a priming strategy would augment therapeutic effects of robot-assisted therapy. Thirty-seven chronic stroke survivors (24 male/13 female; age = 49.8 ± 13.7 years) were randomized to receive mirror therapy or sham mirror therapy prior to robot-assisted therapy. All participants received 18 intervention sessions (60 min/session, 3 sessions/week). Outcome measures were evaluated at baseline and after the 18-session intervention. Motor function was assessed using Fugl-Meyer Assessment and Wolf Motor Function Test. Daily function was assessed using Nottingham Extended Activities of Daily Living Scale. Self-efficacy was assessed using Stroke Self-Efficacy Questionnaires and Daily Living Self-Efficacy Scale. Data was analyzed using mixed model analysis of variance. Both groups demonstrated statistically significant improvements in measures of motor function and daily function, but no significant between-group differences were found. Participants who received mirror therapy prior to robot-assisted therapy showed greater improvements in measures of self-efficacy, compared with those who received sham mirror therapy. Our findings suggest that sequentially combined mirror therapy with robot-assisted therapy could be advantageous for enhancing self-efficacy post-stroke.

**Trial registration**: ClinicalTrials.gov Identifier: NCT03917511. Registered on 17/04/2019, https://clinicaltrials.gov/ct2/show/ NCT03917511.

## Introduction

Stroke is a leading cause of death and long-term disability^1^. Upper limb paresis is a common sequela of stroke, which affects more than 80% of stroke survivors in acute phase and more than 40% in chronic phase^2^. Given the high incidence of upper limb impairments after stroke, restoration of upper limb function has been identified as a top research priority from stroke survivors, caregivers, and health professionals' perspectives^3^. Identifying effective intervention strategies for improving upper limb function is critical in rehabilitation since impairments of upper limb significantly affect the performance of activities of daily living and quality of life^4^.

Robot-assisted therapy is an effective intervention for promoting upper limb function in stroke survivors since robot-assisted therapy provides patients with intense, repetitive practice which is considered a key element for motor training^5,6^. Due to the importance of hand function that accounted for most of delicate movements in daily activities^7,8^, a growing number of robotic devices have been developed to emphasize distal arm training^9^. Systematic reviews of robot‐assisted arm training after stroke showed that the intervention led to improvements in upper limb function, muscle strength and activities of daily living^9–11^. While robot‐assisted therapy is comparable to conventional therapy, combining robot‐assisted therapy with other rehabilitation programs has been suggested as a more effective approach in upper limb rehabilitation^12–14^. Priming techniques are potential methods that can be combined with robot-assisted therapy to augment therapeutic effects. Priming the brain leads to modulation of cortical excitability, which may create a beneficial environment for neurons to reorganize in response to therapy and facilitate neuroplasticity^15,16^. Priming can be implemented through exposure to specific stimuli, such as non-invasive brain stimulation, somatosensory stimulation, motor imagery and action observation^17,18^. In this study, we used a motor imagery and action observation-based priming technique, called mirror therapy, as a priming method prior to subsequent robot-assisted therapy.

Mirror therapy is an easy-to-use and cost-effective intervention in neurorehabilitation and it has been shown to improve motor function of the upper limb in stroke survivors^19^. In mirror therapy, a mirror is placed between the two arms with the reflective side faces the non-paretic side. Patients perform bilateral arm movements and perceive the visual illusion of the paretic arm's movements by observing the reflection of the non-paretic arm’s movements^20^. The mirror-induced visual illusion could facilitate neural activities in motor-associated network of the brain^21^ and therefore serve as a priming technique for inducing neuroplasticity^22^. To our knowledge, only one study examined the effects of sequential combination of mirror therapy and robot-assisted therapy on upper limb motor recovery in patients with stroke^23^. Rong et al. found that subacute stroke survivors who received mirror visual feedback priming prior to proximal-emphasized robot-assisted training showed greater improvements in Fugl-Meyer Assessment for Upper Extremity than those who received sham mirror visual feedback prior to robot-assisted training^23^. No study attempted to combine mirror therapy with distal-emphasized robot-assisted therapy in chronic stroke survivors where function of distal part of upper arm is critical for daily life.

The goals of stroke rehabilitation are not only to improve motor function but also to help stroke patients regain independence and daily life participation. Limitation of activity participation may cause adverse effects on life satisfaction and affect quality of life^24^. In this study, we selected daily function as one of the outcome measures to examine whether participants could translate the improvements of motor function to daily activity performance. We expected that participants would exhibit greater independence in activities of daily living after receiving the intervention program. Apart from motor function and daily function, self-efficacy has been identified as a predictor of rehabilitation outcomes for stroke patients^25^. Self-efficacy is defined as the degree of confidence in one's ability to successfully perform a task^26^. Studies showed that self-efficacy correlates positively with mobility, independence in daily living and quality of life, and correlates negatively with depression in patients with stroke^27–31^. Since self-efficacy has been shown to influence the recovery of stroke patients, we examined whether participants could improve self-efficacy after receiving the intervention program.

The purpose of this study was to examine whether mirror therapy would augment therapeutic effects of robot-assisted therapy on motor function, daily function, and self-efficacy in chronic stroke survivors. Mirror therapy was applied prior to robot-assisted therapy as a priming technique and sham mirror therapy with robot-assisted therapy was used as a control condition in this study. We hypothesized that sequential combination of mirror therapy and robot-assisted therapy would lead to greater improvements in the objective and subjective health-related outcomes than sham mirror therapy with robot-assisted therapy.

## Methods

### Study design and participants

This study was a single-blinded, randomized controlled trial to investigate whether using mirror therapy as a priming strategy would augment therapeutic effects of robot-assisted therapy on motor function, daily function, and self-efficacy in chronic stroke survivors (ClinicalTrials.gov Identifier: NCT03917511, registered on 17/04/2019). The institutional review boards of Chang Gung Memorial Hospital approved the trials (IRB No. 201801025B0C603), and all participants provided written informed consent before participating. All methods were performed in accordance with relevant guidelines and regulations. The sample size of this study was estimated based on the systematic review and meta-analysis of robot-assisted therapy on upper limb recovery after stroke, which showed medium to large effect sizes measured by Fugl-Meyer Assessment^32–34^. We conducted a priori power analysis for repeated measures within-factor test using G*Power software (G*Power 3.1.9.7) to estimate our sample size requirement^35,36^. We found that a total of 36 participants will be required (18 participants in each group) for a medium to large effect size (Cohen's f = 0.35) with a power of 0.8 and type I error of 0.05. Considering an estimated 10 ~ 15% dropout rate, we recruited 43 participants in this study.

**Figure 1:**
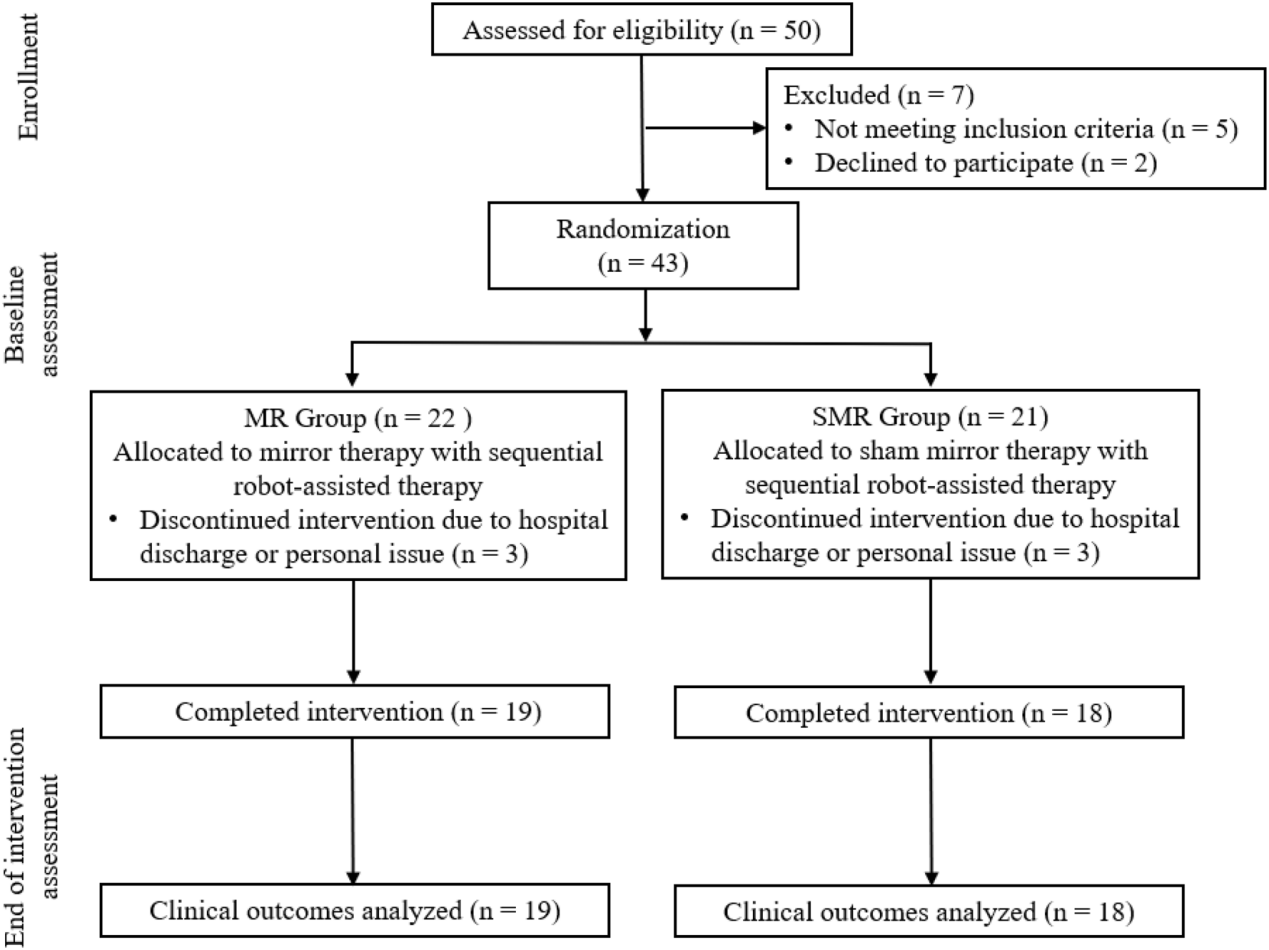
Flow diagram illustrating the flow of participants through each stage of the study.

Participants were recruited from medical centers in Taiwan, who attended for post-stroke rehabilitation between December 2018 and April 2021. The inclusion criteria included: (1) unilateral stroke ≥ 3 months prior to study enrollment; (2) Fugl-Meyer Assessment for Upper Extremity (FMA-UE) score < 60; (3) without excessive spasticity in any of the UE joint (modified Ashworth scale ≤ 3); (4) Mini Mental State Exam (MMSE) score > 24, indicating no serious cognitive impairment; and (5) between the ages of 20 and 75 years. The exclusion criteria included: (1) histories of other neurological diseases such as dementia and peripheral polyneuropathy; (2) difficulties in following and understanding instructions such as global aphasia; (3) enroll in other rehabilitation or drug studies simultaneously; (4) receiving Botulinum toxin injections within 3 months. The research design and flow process are shown in Fig. 1.

### Intervention protocol

Participants were stratified into four strata based on the lesion side (left and right) and the initial upper extremity impairment levels (Fugl-Meyer Assessment for Upper Extremity score < 35 and ≥ 36)^37^, and randomly allocated to either the mirror priming group (MR) or sham mirror priming group (SMR). An investigator who was not involved in the evaluation and treatment managed the randomization procedure by using a random-number table generated online (freely available at http://www.randomizer.org/). All participants received interventions for 60 min/session, 3 sessions/week for 6 consecutive weeks. In MR group, each intervention consisted of 20 min of mirror therapy, followed by 40 min of robot-assisted therapy. In SMR group, each intervention consisted of 20 min of sham mirror therapy, followed by 40 min of robot-assisted therapy. All interventions were conducted by a certified occupational therapist.

#### Mirror therapy protocol

A mirror was placed in the participants’ midsagittal plane to create a visual illusion of a paretic limb by using the mirror reflection of the non-paretic arm. The paretic arm was placed behind a mirror without being seen by the participant. During the 20-min mirror therapy, a robotic hand was attached to the paretic hand and provided continuous passive motion including 10-min grasp and release motion and 10-min pinch and release motion. Participant were instructed to look at the reflection of the non-paretic arm in the mirror, imagined it as the paretic arm and perform bilateral hand movements as symmetrically as possible. For the sham mirror priming group, participants underwent the same protocol except the mirror was covered with black fabric.

#### Robot-assisted therapy protocol

The 40-min robot-assisted therapy consisted of 10-min active-assisted training and 30-min interactive training using the Hand of Hope (HOH) robotic hand system (Rehab-Robotics Co. Ltd, Hongkong, China). HOH is an exoskeleton type of robot with 2 surface electromyography (EMG) sensors that detect the level of motor unit recruitment. In active-assisted training, the robot provided participants with assistive movements that guided the fingers to complete grasp and release motion or pinch and release motion, once the EMG signal exceeds the predetermined threshold. In interactive training, the therapist selected 3 interactive games from the robot system and chose the level of difficulty based on participants' upper limb functional status. Participants were instructed to coordinate arm and hand movements to complete game missions. A detailed description of the robot was presented in a previous paper^38^.

### Outcome measurements

We used clinical assessments to examine three domains of therapeutic effects of sequential combination of mirror therapy and robot-assisted therapy: (1) motor function, (2) independence in daily function, and (3) self-efficacy. Clinical assessments included Fugl-Meyer Assessment for Upper Extremity (FMA-UE), Wolf Motor Function Test (WMFT), Nottingham Extended Activities of Daily Living Scale (NEADL), the stroke self-efficacy questionnaires (SSEQ) and Daily Living Self-Efficacy Scale (DLSES). Participants were assessed within 1 week before the intervention (baseline assessment), and after the 18-session intervention (post-assessment). All participants were assessed by a certified occupational therapist who was unaware of the group to which the participant had been allocated.

#### Domain of motor function

Fugl-Meyer Assessment for Upper Extremity (FMA-UE): The FMA-UE includes 33 items assessing movements, reflexes, and coordination of upper limbs. Each item is measured on a 3-point ordinal scale and the total score ranges from 0 to 66^39^. A higher score indicates better motor function. The reliability and validity of the Fugl-Meyer Assessment are well established^7,40^.

Wolf Motor Function Test (WMFT): The WMFT assesses upper extremity motor ability by measuring the performance time (WMFT-Time) and functional ability rating scale (WMFT-FAS) in required task. Participants were timed and rated by using a 6-point ordinal scale. The WMFT is valid and reliable in assessing motor function in stroke patients^41,42^.

#### Domain of independence in daily function

Nottingham Extended Activities of Daily Living Scale (NEADL): The NEADL is a measure of independence in 4 areas of daily life, including mobility, kitchen, domestic, and leisure activities. It includes 22 items, and each item is measured on a 4-point scale. The total score ranges from 0 to 66 and a higher score indicates better daily functional ability. The psychometric properties of the NEADL have been well established^43^.

#### Domain of self-efficacy

The stroke self-efficacy questionnaires (SSEQ): The SSEQ measures an individual's confidence in relation to functional performance and self-management after stroke. It includes 13 items, and each item is rated on a 10-point scale from 0 (not at all confident) to 10 (very confident). The reliability and validity of the SSEQ are well established^44^.

Daily Living Self-Efficacy Scale (DLSES): The DLSES measures self-efficacy of daily functioning, including psychosocial functioning and activities of daily living. The scale consists of 12 items, and each item is measured on a 100-point scale with 10-unit intervals (0 = cannot do at all, 100 = highly certain can do). A higher score indicates higher level of self-efficacy. The DLSES is a psychometrically sound measure of self-efficacy in stroke survivors^45^.

### Statistical analysis

The *Chi-*square tests and independent *t*-tests were used to compare participants' baseline demographic and clinical characteristics between two groups. We used mixed model analysis of variance (ANOVA) to examine treatment effects between interventions (mirror therapy with robot-assisted therapy vs. sham mirror therapy with robot-assisted therapy). We defined "intervention" as the within-subjects factor (before and after intervention) and "group" as the between-subjects factor (mirror priming group vs. sham mirror priming group). Partial eta squared (η_p_^2^) was computed for each variable as a measure of effect size. For all calculations, a significance level at α = 0.05 was used. All tests were executed using the SPSS software version 25 (International Business Machines Corp., Armonk, NY).

## Results

### Demographic characteristics of both groups

We screened 50 patients for eligibility. Forty-three of them met the inclusion criteria and were randomly assigned to two groups. During the intervention period, six participants withdrew from the study, and they were excluded from data analysis (Fig. 1). There were 19 participants in the MR group and 18 participants in SMR group. Descriptive characteristics of participants are presented in Table 1. The two groups did not differ significantly in terms of participants' demographic and clinical characteristics.

**Table 1:** Demographic characteristics and clinical background of participants.

Variables	MR Group (N = 19)	SMR Group (N = 18)	*p*-value
Gender (Male/Female)	13/6	11/7	0.64
Affected side (R/L)	13/6	7/11	0.07
Age (years), Mean ± SD	47.68 ± 14.28	52.0313.07	0.34
Time since stroke (months), Mean ± SD	37.50 ± 38.63	26.25 ± 24.58	0.55
FMA-UE, Mean ± SD	34.58 ± 12.84	35.00 ± 11.22	0.92
MMSE, Mean ± SD	27.32 ± 2.56	27.89 ± 2.74	0.52

MR Group = Mirror therapy with robot-assisted therapy; SMR Group = Sham mirror therapy with robot-assisted therapy.

*FMA-UE* Fugl-Meyer Assessment for upper extremity, *MMSE* Mini Mental State Exam.

### Motor function

The mean and standard deviation for clinical outcome measures were shown in Table 2. Results of inferential statistics were shown in Table 3. Results of FMA-UE showed no statistically significant interaction between groups and intervention (*p* = 0.39). For the main effect, we found a statistically significant effect of intervention F(1,35) = 31.57, *p* < 0.005, η_p_^2^ = 0.47, and there was no statistically significant effect of group (*p* = 0.82).

**Table 2:** Descriptive statistics for clinical outcome measures.

Outcome measure	Baseline (N = 37)		End of intervention (N = 37)	
	MR group	SMR group	MR group	SMR group
FMA-UE	34.58(12.84)	35.00(11.22)	37.42(13.38)	38.89(11.69)
WMFT-FAS	2.79(0.97)	2.59(0.81)	2.92(0.83)	2.75(0.74)
WMFT-Time	14.10(7.79)	12.83(8.52)	15.00(9.15)	13.15(7.05)
NEADL	28.26(17.83)	26.89(15.08)	32.89(16.38)	31.83(14.68)
SSEQ	92.58(16.98)	78.06(26.54)	99.21(13.89)	85.61(23.33)
DLSES	60.48(24.03)	53.03(25.51)	72.19(19.39)	57.41(24.73)

Data are presented as the mean (SD).

MR Group = Mirror therapy with robot-assisted therapy; SMR Group = Sham mirror therapy with robot-assisted therapy.

*FMA-UE* Fugl-Meyer Assessment for upper extremity, *WMFT-FAS* functional ability scale of the Wolf Motor Function Test, *WMFT-Time* performance time of the Wolf Motor Function Test, *NEADL* Nottingham Extended Activities of Daily Living Scale, *SSEQ* Stroke Self-Efficacy Questionnaire, *DLSES *Daily Living Self-Efficacy Scale.

**Table 3:** Inferential statistics for outcome measures.

Outcome measure	Effect	*df*	F	*p*	η_p_^2^
FMA-UE	Within subjects				
	Intervention	1	31.57	< 0.005*	0.474
	Intervention × group	1	0.76	0.39	0.021
	Error	35			
	Between subjects				
	Group	1	0.06	0.82	0.002
	Error	35			
WMFT-FAS	Within subjects				
	Intervention	1	14.59	0.001*	0.294
	Intervention × group	1	0.71	0.602	0.008
	Error	35			
	Between subjects				
	Group	1	0.462	0.501	0.013
	Error	35			
WMFT-Time	Within subjects				
	Intervention	1	0.447	0.508	0.013
	Intervention × group	1	0.1	0.753	0.003
	Error	35			
	Between subjects				
	Group	1	0.382	0.541	0.011
	Error	35			
NEADL	Within subjects				
	Intervention	1	9.119	0.005*	0.207
	Intervention × group	1	0.01	0.922	< 0.005
	Error	35			
	Between subjects				
	Group	1	0.058	0.811	0.002
	Error	35			
SSEQ	Within subjects				
	Intervention	1	9.628	0.004*	0.216
	Intervention × group	1	0.041	0.841	0.001
	Error	35			
	Between subjects				
	Group	1	4.826	0.035*	0.121
	Error	35			
DLSES	Within subjects				
	Intervention	1	7.913	0.008*	0.184
	Intervention × group	1	1.646	0.208	0.045
	Error	35			
	Between subjects				
	Group	1	2.4	0.13	0.064
	Error	35			

Main effects of within-subject factor (intervention) and between-subjects factor (group), along with interactions.

*p value < 0.05.

As for the results of WMFT-FAS, there was no statistically significant interaction between groups and intervention (*p* = 0.60). For the main effect, we found a statistically significant effect of intervention F(1,35) = 14.59, *p* = 0.001, η_p_^2^ = 0.29, and there was no statistically significant effect of group (*p* = 0.50). Results of WMFT-Time showed no statistically significant interaction between groups and intervention (*p* = 0.75). For the main effect, there was no statistically significant effect of intervention (*p* = 0.51) and no statistically significant effect of group (*p* = 0.54).

### Daily function

Results of NEADL showed no statistically significant interaction effect between group and intervention (*p* = 0.92). For the main effect, there was a statistically significant effect of intervention, F(1,35) = 9.12, *p* = 0.005, η_p_^2^ = 0.21, and there was no statistically significant effect of group (*p* = 0.81). Participants in both groups significantly improved their independence in daily activities, and the improvements were not significantly different between the two groups.

### Self-efficacy

Results of SSEQ showed no statistically significant interaction effect between group and intervention (*p* = 0.84). For the main effects, the results revealed a statistically significant effect of intervention, F(1,35) = 9.63, *p* < 0.005, η_p_^2^ = 0.22, and a statistically significant effect of group, F(1,35) = 4.83, *p* = 0.035, η_p_^2^ = 0.12. Since there were no significant difference in baseline measures of SSEQ between two groups, *t*(35) = 1.994, *p* = 0.054, we then conducted pairwise comparisons to examine the differences between baseline and post-intervention for each group. We found significant improvements in SSEQ scores from baseline to post-intervention in MR group, *t*(18) = 2.35, *p* = 0.03, and no significant differences were found in SMR group, *t*(17) = 2.08, *p* = 0.053.

Results of DLSES showed no statistically significant interaction effect between group and intervention (*p* = 0.21). For the main effects, the results revealed a statistically significant effect of intervention, F(1,35) = 7.91, *p* = 0.008, η_p_^2^ = 0.18, and there was no statistically significant effect of group (*p* = 0.13). Since there were no significant difference in baseline measures of DLSES between two groups, *t*(35) = 0.915, *p* = 0.366, we then conducted pairwise comparisons to examine the differences between baseline and post-intervention for each group. We found significant improvements in DLSES scores from baseline to post-intervention in MR group, *t*(18) = 2.37, *p* = 0.03, and no significant differences were found in SMR group, *t*(17) = 1.62, *p* = 0.12.

## Discussion

In this study, we used a randomized controlled trial to examine whether mirror therapy would augment therapeutic effects of robot-assisted therapy on motor function, daily function, and self-efficacy in chronic stroke survivors. Based on the timeline of stroke recovery framework proposed by SRRR group, chronic stroke is defined as 6 months after stroke onset and 3 to 6 months post-stroke refers to as late sub-acute phase^46^. In stroke rehabilitation studies, several researchers used chronic stroke to describe patients who were at least 3 months after stroke^47,48^ and we adopted the broad definition of chronic stroke in this study. Our findings indicated that chronic stroke survivors seem to benefit from robot-assisted therapy in upper limb function and functional independence. Applying mirror therapy prior to robot-assisted therapy could further improve self-efficacy for stroke patients. Although mirror therapy did not significantly augment the effects of robot-assisted therapy on objective measures of upper limb function, stroke patients who received mirror therapy prior to robot-assisted therapy significantly improved their confidence in performing daily activities.

In motor function domain, results of FMA-UE and WMFT-FAS demonstrated the positive effects of robot-assisted therapy on upper limb motor recovery and functional performance, and the effect size of intervention was large based on benchmarks suggested by Cohen^49^. However, applying mirror therapy prior to robot-assisted therapy did not augment the therapeutic effects as the improvements were not significantly different between the MR and SMR groups. Considering the effect size estimation, a large effect size suggested that robot-assisted therapy produced a significant impact on improving motor function and therefore mirror therapy might make a relatively small impact on augmenting therapeutic effects on motor function domain.

In contrast to improvements in FMA-UE and WMFT-FAS scores, participants did not improve their performance time for completing the tasks after the interventions in both groups based on the results of WMFT-Time scores. One explanation is that movement speed was not the primary focus of our intervention in this study. Our robot-assisted therapy consisted of 10-min active-assisted training and 30-min interactive training. The primary focus of active-assisted training was to assist patients in precisely recruiting the desired muscle group and enhance muscle activation during functional movements. Additionally, interactive training emphasized on coordination of arm and hand movements to improve endpoint accuracy during functional tasks. Participants were not asked to move in a fast pace during training and therefore the speed of task performance did not significantly improve. While temporal efficiency is identified as an aspect of movement quality^50^, future studies can investigate the effect of adopting speed-focused training to improve the effectiveness of robot-assisted therapy.

Our results in motor function domain seemed to conflict with the findings from Rong et al.'s study, which showed mirror therapy could augment therapeutic effects of robot-assisted therapy in motor recovery measured by FMA-UE^23^. One explanation is that a time window of heightened neural plasticity might exist following stroke, leading to enhanced responsiveness to priming technique and training^51^. Stroke patients in subacute stage might be more responsive to than chronic stroke survivors in terms of augmenting the effect of robot-assisted therapy in motor function domain. Nevertheless, we found that chronic stroke survivors could benefit from distal-emphasized robot-assisted therapy and significantly improve upper limb motor function.

In daily function domain, our findings showed that participants successfully transferred gains in upper limb motor function to daily functional ability after robot-assisted interventions, measured by NEADL. Regarding our robot-assisted intervention protocols, active-assisted training mainly focused on neuromuscular control of hand movement while interactive training required coordinated motions for arm and hand. Integration of distal and proximal upper limb training has been advocated as a key for enhancing functional gains^52^. Moreover, interactive training adapted the concepts of task-oriented training through interactive games that mimic real-life tasks such as gardening. As a result, participants might easily transfer improvements in upper limb motor function to functional activities in daily life.

Limited research has been conducted to identify stroke survivors' self-efficacy after receiving robot-assisted therapy using standardized scales. We used two standardized scales to measure self-efficacy since they capture different aspects of self-efficacy in stroke population. Whereas SSEQ was developed to measure one's confidence in relation to functional performance following stroke, DLSES captures self-efficacy in a broader sense including psychosocial functioning and activities of daily living. Results of both measures suggest that applying mirror therapy prior to robot-assisted therapy could be advantageous for enhancing self-efficacy post-stroke. Applying mirror therapy prior to robot-assisted therapy led to significant improvements in stroke survivors' self-efficacy based on the results of SSEQ and DLSES scores. According to Bandura's self-efficacy theory, self-efficacy can be developed by four main sources of influence, including mastery experience , vicarious experience, verbal persuasion, and emotional arousal^53^. Mastery experience and vicarious experience are two factors that may explain the effects of mirror therapy on self-efficacy. During mirror therapy, participants could obtain vicarious experience by observing the reflection of non-paretic arm movements and imagining as if it were the paretic arm performing movements. Moreover, participants were instructed to perform symmetrical hand movements during mirror therapy. The robotic hand guided the paretic hand to successfully execute movements, which could contribute to the achievement of mastery experience. Hence, stroke survivors can strengthen their self-efficacy by integrating mirror therapy into upper limb rehabilitation program.

This study has some limitations. First, there were more participants with left hemisphere damage in MR group and more participants with right hemisphere damage in SMR group. Although there were no statistically significant differences in participants' demographic and clinical characteristics between the MR and SMR groups, side of hemispheric lesions could affect stroke rehabilitation in upper limb training^54^. Future studies could examine whether the side of hemispheric lesions influence priming effect of mirror therapy and therapeutic effect of robot-assisted therapy. Second, we recruited chronic stroke survivors who were at least three months after the onset of stroke in our study. Therefore, caution should be exercised when attempting to generalize our findings to stroke patients in acute or subacute stage. Third, only the assessor was blinded to participants' intervention allocation in this study. Although the blinding of participants and therapists is nearly impossible for most rehabilitation trials in occupational therapy, lack of blinding could become a risk of bias associated with non-blinded participants and therapists who conducted the intervention. Since all participants underwent the same training protocol during robot-assisted therapy and the therapist chose the level of difficulty based on participants' upper limb functional status, the impact of risk of bias may be limited. Fourth, the sample size estimation was based on the effect size of robot-assisted therapy on upper limb motor recovery after stroke without taking into account the effect size of primming strategies in motor recovery. We might underestimate the required sample size for this study to observe the significant impact of mirror therapy on augmenting therapeutic effects on motor function domain. Lastly, we used an exoskeleton robotic hand to perform interventions in this study. Studies have shown that exoskeleton robots could be more effective in treating stroke patients with more severe motor impairments and patients in the subacute stage, whereas end-effector robots could be more effective in treating patients with mild-to-moderate motor impairments and patients in the chronic stage^55,56^. Future studies could examine whether exoskeleton robots and end-effector robots engender different therapeutic effects among patients with different degrees of motor impairment while applying mirror therapy as a priming strategy.

## Conclusion

Robot-assisted therapy and mirror therapy are both effective in promoting upper limb function after stroke and combining these two interventions was hypothesized to yield greater therapeutic effects. Our findings indicated that chronic stroke survivors seem to benefit from robot-assisted therapy in upper limb function and functional independence. Applying mirror therapy prior to robot-assisted therapy could further improve self-efficacy for stroke patients. While mirror therapy did not augment the effects of robot-assisted therapy on domain of motor function and independence in daily function, future studies are needed to investigate the effect of applying alternative priming technique prior to robot-assisted therapy, such as non-invasive brain stimulation, to improve the effectiveness of robot-assisted intervention for patients with stroke.

## Data Availability

The datasets used and/or analyzed during the current study are not publicly available due to the confidentiality issue but are available from the corresponding author upon reasonable request.
